# Task demand and load carriage experience affect gait variability among military cadets

**DOI:** 10.1038/s41598-022-22881-y

**Published:** 2022-11-01

**Authors:** Sophia Ulman, Divya Srinivasan, Maury A. Nussbaum

**Affiliations:** 1 Scottish Rite for Children, Frisco, TX USA; 2 Department of Industrial and Systems Engineering, Virginia Tech, Blacksburg, VA USA; 3 Present Address: Department of Industrial Engineering, Clemson University, 277A Freeman Hall, Clemson, SC 29634 USA

**Keywords:** Biomedical engineering, Musculoskeletal system

## Abstract

Load carriage is an inevitable daily task for soldiers. The purposes of this study were to explore the extent to which gait variability (GV) is affected by load carriage and experience among military cadets, and whether experience-related differences in GV are dependent on task demand. Two groups of cadets (30 experienced, 30 less experienced) completed a load carriage task in each of three load conditions (no load, 16 kg, 32 kg). Three categories of GV measures were obtained: spatiotemporal variability, joint kinematic variability, and Lyapunov exponents. Compared to traditional mean gait measures, GV measures were more discriminative of experience: although both groups showed similar mean gait measures, the experienced participants had reduced variability in spatiotemporal measures (*p* ≤ 0.008) and joint kinematics (*p* ≤ 0.004), as well as lower levels of long-term local dynamic stability at the ankle (*p* = 0.040). In both groups, heavier loads were also caused increased GV (*p* ≤ 0.018) and enhanced short-term local dynamic stability at the knee (*p* = 0.014). These results emphasize the importance of GV measures, which may provide a more complete description of adaptability, stability, and control; highlight alternate movement strategies during more difficult load carriage; and capture experience-related differences in load carriage strategies.

## Introduction

Load carriage is an inevitable daily task for dismounted soldiers in combat environments^1^. On duty, soldiers must transport their equipment in a rucksack, which can require carrying an additional load of approximately 10 to 60% of their body weight^2^, and they must often do so while also maintaining a high level of performance^3,4^. The added load, often carried in extreme environments, can lead to substantial fatigue, hinder performance, and increase injury risk^5^. Consequently, considerable research has been done to evaluate the biomechanical changes in gait that result from carrying substantial loads^6–10^. Some studies have yielded conflicting findings on gait spatiotemporal measures. For example, Krupenevich et al. found a reduced stride length in loaded verses unloaded gait, while others have reported that additional load has no effect on spatiotemporal measures^6,7,9^. In terms of lower-limb joint kinematics, load carriage has most commonly been found to increase sagittal hip and ankle range-of-motion^2,8^. Existing research and evidence on gait characteristics (kinetics and kinematics) during load carriage has contributed to enhancing the effectiveness of equipment design and assessment, developing load carriage techniques for military populations, decreasing task difficulty and injury risk, and improving efficiency^11^.

However, load carriage continues to be one of the most common causes of overuse injuries to the lower limbs for dismounted soldiers^12,13^. Additionally, while the range of experience among active-duty Army soldiers can exceed 20 years^14^. it is still unclear to what extent experience influences gait strategies utilized for load carriage. Effects of experience level is especially relevant as injuries related to load carriage are particularly high during basic training, when cadets experience large increases in habitual training load^14^. and such injuries often persist long after basic training^15,16^. Hence, it is important to understand how experience affects load carriage movement strategies and whether specific movement strategies may be associated with lower injury risks due to load carriage. In the military domain, higher levels of experience have been shown to positively affect psychophysiological responses during a parachute jump^17^ and urban combat^18^. Load carriage experience has also been associated with faster road march times^19^ and an increased ability to carry heavy loads^20^. However, specific movement patterns that contribute to increased load carriage performance in more experienced military personnel remain unclear.

Human gait is an inherently complex activity, requiring the control and coordination of many neurophysiological and biomechanical degrees-of-freedom. Successful gait often requires altering locomotor patterns in response to changing environmental or task demands^21^. Gait variability (GV) is a fundamental feature of gait that refers to the variation observed in the spatiotemporal dispersion of joint movements, inter-joint coordination patterns, and muscle activities. While it is important to know how load influences average (or typical) gait characteristics, measures of variability provide a more complete description of an individual’s adaptability, stability, and control throughout challenging task conditions^22–24^. For example, increased spatiotemporal GV corresponds with an increase in fall risk among older adults^24,25^. Similarly, individuals with chronic low back pain exhibit increased GV in a dual-task condition compared to healthy individuals, indicating that chronic pain may hinder motor-cognitive skills^26^. During load carriage in healthy populations, carrying heavier load leads to increased step width variability^23,27^, unaltered joint angle variability^23^, and increased variability in trunk-thigh coordination in the sagittal and transverse planes^22,23^. These reported changes in GV have been hypothesized to correspond with an increase in adaptability and stability, suggesting that altered movement strategies may result from an individual adjusting to the additional load during gait, potentially to reduce injury risk. However, additional research is needed to quantify the extent to which GV is affected by increased loads and/or experience level in the military domain.

In summary, experience level could have a substantial influence on the movement strategies used by soldiers during load carriage, and GV could be useful for quantifying this influence. Specifically, more complex measures of GV are expected to yield a better understanding of how experience level impacts load carriage gait and aid in identifying specific movement strategies that support task performance. Furthermore, identifying effective ranges of GV in diverse military populations could help establish normative bounds on movement patterns that can guide the development of load carriage equipment such as rucksacks, or assistive/augmentation systems (e.g., exoskeletons). GV was quantified here using three categories of measures, which included spatiotemporal variability, joint angle variability, and Lyapunov exponents. The current study was exploratory and is, to our knowledge, the first to examine the relationship between experience level and GV during a military-relevant load carriage task, in which multiple load conditions were considered. Based on prior evidence showing that individuals with various skill levels exhibit different levels of variability^28–31^, we anticipated that GV would differ between two groups with distinct levels of load carriage experience, and that this difference would be more pronounced with higher task demands (higher loads).

## Methods

### Experimental design and participants

A mixed-factor design was used to test the effects of two independent variables, experience level (between-subjects factor) and load condition (within-subjects factor), on gait variability. First-year and fourth-year Reserve Officers’ Training Corps cadets at a local university were recruited to represent two different levels of military-relevant load carriage experience. Specifically, by the time of testing the 4th-year cadets had performed weekly load carriage training during the three prior academic school years; they had also completed a mandatory advanced training camp for 6 weeks during the summer prior to their 4th year that involved extensive load carriage training. In contrast, 1st-year cadets were considerably less experienced, as they had only completed a semester of training prior to participation in this study. Additionally, to ensure the 1st-year cadets tested were indeed less experienced, any cadet who had completed additional load carriage training outside of their standard weekly training requirements (in the 1st year group) was excluded from participation.

A total of 60 1st- and 4th-year cadets (30 in each group) completed the study, and all participants provided informed consent. The study protocol was conducted according to the Declaration of Helsinki. Study procedures were approved by the Virginia Tech Institutional Review Board (Approval ID #18-800) and performed in accordance with the relevant guidelines and regulations. No participants reported having any current or recent history of musculoskeletal disorders or injuries. Age, stature, body mass, and fitness level were collected (Table 1), the latter determined using standardized Army Physical Fitness Test (APFT) scores^32^. Cadets reported their score from the most recent APFT (one score was not received from a 1st-year cadet). Results from unpaired *t* tests indicated that there were no significant differences between the two groups (1st- and 4th-year cadets) in stature (*p* = 0.201) or fitness level (*p* = 0.102); however, the 4th-year group had a significantly higher body mass (*p* = 0.027).

**Table 1:** Demographic and anthropometric data.

Participant group	*n*	Age (years)	Stature (m)	Body Mass (kg)	APFT score
**1st-year cadets**	30	18.5 (0.5)	1.8 (0.1)	76.7 (10.9)	259.6 (23.1)
Female	4	18.0 (0.5)	1.7 (0.1)	75.6 (16.8)	279.0 (29.8)
Male	26	18.5 (0.5)	1.8 (0.1)	77.6 (10.0)	258.8 (23.2)
**4th-year cadets**	30	21.9 (0.7)	1.8 (0.1)	83.5 (12.3)	270.1 (25.8)
Female	7	22.3 (1.0)	1.6 (0.1)	70.9 (10.7)	263.0 (32.0)
Male	23	21.8 (0.6)	1.8 (0.1)	86.8 (10.5)	271.9 (24.4)

All values reported as means (SDs).

Three load conditions were tested: no load (Load_Zero_), light load (Load_Low_), and heavy load (Load_High_). Based on a review of load carriage studies testing both civilians and military personnel^2^, the two loaded conditions were chosen to simulate typical loads tested in military-related studies. Additionally, training for the Reserve Officers’ Training Corps requires cadets to be familiar with carrying considerable loads for long periods of time. This training begins immediately in their first academic year and consists typically of weekly or biweekly marches with a 16 kg load for distances of 7–20 km. Given the level of familiarity each cadet would then have with this standard load, we thought that normalizing the load (i.e., as a percentage of body mass) would likely alter the natural kinematics established from the cadets’ regular training. We aimed to create a testing protocol that was similar to their typical training loads, to the extent possible, so as to successfully capture typical behaviors and movement patterns. Thus, the Load_Low_ condition was 16 kg, which was equivalent to 21% and 19% of mean 1st- and 4th-year cadet body mass, respectively.

To be consistent, we also had all cadets carry the same heavy load. While we again considered normalizing the heavier load, this approach would lead to a situation that was not reflective of common practice within this population. Prior to their fourth year of training, all cadets (irrespective of their body mass) were required to complete a summer boot camp that includes 9.5, 13, and 20 km hikes with higher loads (up to 32 kg). Specifically, all cadets are required to carry the same load regardless of their height or body mass. Thus, all 4th year cadets tested, regardless of anthropometry, were equally trained and familiar with the heavy load condition used here. Further, we did not believe that, for example, it would be appropriate for a larger male to carry a heavier load or a smaller female to carry a lighter one, given expectations in the military and their equal level of familiarity with the 32 kg load condition. Thus, the Load_High_ condition was 32 kg, or 42% and 38% of mean 1st- and 4th-year cadet body mass, respectively. The order of load conditions was counterbalanced using multiple 3 × 3 Balanced Latin Squares.

### Procedures and data collection

All participants were asked to wear athletic attire, including shorts and their own military marching boots, and to use their personal rucksack during testing. For load carriage, many studies referenced in an earlier review^2^ used a single rucksack with a rigid frame, and the most common military-grade rucksack used for testing was the Modular Lightweight Load-carrying Equipment^10,22,33^. This specific pack was issued to all cadets for load carriage tasks and exercises and was therefore easily available for testing. Prior to data collection, the straps of the pack and the position of the frame were adjusted as needed to ensure a snug and comfortable fit. For the load conditions, packs were loaded with metal plates secured to the pack frame by the experimenters, to eliminate any potential advantage related to packing strategies across all cadets.

**Figure 1:**
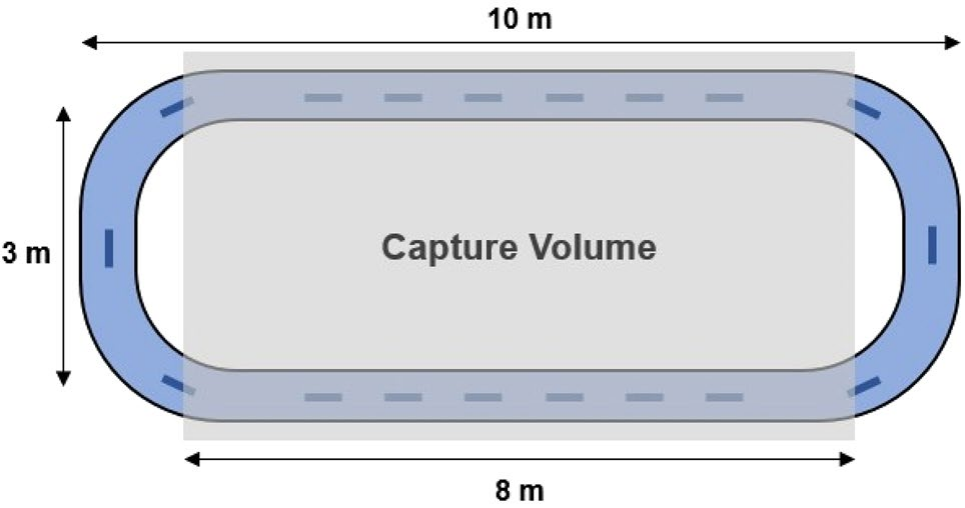
Schematic diagram of the walking path and motion-capture volume (not to scale).

Participants were asked to walk continuously for seven minutes at a comfortable, self-selected pace, since constraining gait speed can influence “natural” GV patterns^34^. Participants were instructed to follow a rectangularly-shaped path with rounded corners that was marked with tape on the floor of a laboratory (Fig. 1). The walking path consisted of two straight sections that were ~ 10 m long, and two curved sections that were ~ 3 m wide. Walking trials were completed for each load condition, with loads of 0, 16, and 32 kg inserted into the rucksack, and rest periods of 10 min were provided between trials to minimize confounding fatigue effects^2^.

Kinematics of the pelvis and lower limbs were tracked using passive reflective markers placed bilaterally using a modified Helen-Hayes marker set^35^. Triaxial coordinates of these markers were obtained at 120 Hz using a 13-camera motion capture system (Qualisys, INC, Gothenburg, Sweden). Note that for each straight section of the track, roughly 8 m fell within the volume covered by the motion capture system. Kinematics during first lap along the path were not captured, to minimize any potential confounding effects caused by a lack of warm up or familiarity. Only strides within the capture volume, where gait was assumed to be straight and steady, were used for subsequent assessments. At least 50 strides is recommended to reliably calculate GV measures^36^. In the 7-min. duration, a mean (SD) of 92 (26) strides were captured.

### Data processing and outcome measures

Marker data captured from the straight sections of the walking path were extracted. Prior to obtaining linear measures of GV described below, marker data were low-pass filtered (4th-order, recursive, Butterworth, 5 Hz cutoff frequency). Spatiotemporal stride characteristics were computed for further analysis, along with hip, knee, and ankle joint angle time series in the sagittal plane. Given that typical marker placements on the posterior trunk and pelvis were covered by the specific rucksack used for load carriage in this study, trunk kinematics could not be reliably obtained; thus, only lower extremity gait variability was evaluated. In addition, since force plate data were not available, a coordinate-based algorithm was used to determine heel strike and toe-off events^37^. No significant differences were found between the two limbs (from paired *t* tests) for any of the gait measures, and thus only results from the left side were further analyzed. Data from six participants (five 1st-year participants and one 4th-year participant) were excluded due to marker loss during all walking conditions, and data for the Load_Low_ condition was missing for one 4th-year participant due to marker loss.

In addition to mean gait measures, three categories of GV measures were obtained as listed below and described in more detail subsequently. Joint angle variability (JAV), coupling angle variability (CAV), and short- and long-term Lyapunov exponents (SLE/LLE) were all computed from full strides (i.e., the measures do not consider the specific stance or swing phases of the gait cycle).

1. *Spatiotemporal standard deviation (SD)* Measures of statistical dispersion for stride characteristics (stride and step time, step length, and step width).
2. *JAV/CAV* Variability of joint angle kinematics (JAV) at the hip, knee, and ankle, as well as inter-joint coordination variability (CAV) of the hip-knee and knee-ankle joint couples across the full gait cycle.
3. *SLE/LLE* Short- and long-term Lyapunov exponents that quantify local dynamic stability using continuous, joint angle time series data from the hip, knee, and ankle across the full gait cycle.

Means and stride-to-stride standard deviations were calculated for several stride characteristics, which included duration measures (i.e., stride and step time), distance measures (i.e., stride length and step width), and gait speed. Joint angles for the knee and ankle were calculated as Cardan angles between adjacent local segments, with a rotation order of flexion–extension, abduction–adduction, and internal–external rotation^38–40^. Due to the lack of posterior pelvis markers, hip joint angle was defined as the absolute angle of the thigh segment relative to the horizontal. Additionally, only sagittal plane kinematics were analyzed. For joint angle time series, standard deviations were first calculated at each time point (100 points of a normalized gait cycle) across all strides. Then, the overall standard deviation of each joint angle trajectory, or joint angle variability (JAV), was calculated as the root-mean-square of these standard deviations. Inter-joint coordination was quantified using a modified vector coding technique^41,42^ to assess hip-knee and knee-ankle joint coupling angles in the sagittal plane. Coupling angle variability (CAV), the standard deviation across all strides, was calculated for each time point using circular statistics^43^, and then pooled across the gait cycle by computing the overall root-mean-square from all time points. CAV was used as a measure of stride-to-stride coordination variability^44^.

Linear measures, such as spatiotemporal SD, JAV, and CAV, capture the overall magnitude of variation, as statistical dispersions with respect to the mean. Specifically, spatiotemporal SD measures reflect variation in the overall gait pattern, while JAV measures provide more detailed information on variability exhibited at each joint. Furthermore, variability in coordination, or CAV, has been suggested more recently to play an important role in movement adaptation^24,45,46^. Assessing multiple body components in the lower extremities, and how they move in relation to each other, provides a more comprehensive analysis^24^. However, these measures do not capture the time-dependent nature of variability. Thus, they are typically computed along with more advanced measures of variability.

Maintaining stability is essential for load carriage, especially as load increases. Thus, compensatory strategies, or movement variability, may be employed to avoid injury and maintain stability. Gait dynamics include stride-to-stride fluctuations as well as the corresponding variations over time, and some methods for assessing these dynamics stem from chaos theory. To assess the fundamental properties of complex time series, these time series are assumed to be chaotic processes, implying both determinism as well as unpredictability^47^. Measures in this class investigate the embedded structure and nature of movement variability^48^. Specifically, Lyapunov exponents measure the rate of divergence of trajectories within a system. Here, short- and long-term LEs were computed to represent the local dynamic stability of each walking condition at each joint (i.e., hip, knee, ankle) by quantifying the exponential attenuation of variability between neighboring kinematic trajectories^49,50^. This analysis evaluates stride-to-stride differences for a given kinematic measure, such as a joint angle time series, by assuming that every stride could be identical to every other stride in the time series. Positive exponents indicate local instability, while negative (or zero) values indicate a periodic system. Furthermore, larger exponent values indicate faster divergence and increased instability, and thus greater sensitivity to local perturbations^51^. To calculate LE, joint angle times series from the hip, knee, and ankle were filtered using a 2nd-order, low-pass, recursive, Butterworth filter with a cutoff frequency of 10 Hz^49^. Short-term exponents (SLE) were computed between 0 and 1 stride, and long-term exponents (LLE) were computed between 4 and 7 strides^49,50,52^. See Supplementary Methods for additional details. LE exponents could not be derived for four 1st-year and three 4th-year participants.

### Statistical analysis

Separate mixed-factor analyses of covariance (ANCOVAs) were used to assess the effects of experience level (between-subjects) and load condition (within-subjects) on each gait measure, with the order of exposure to each loading conditions included as a blocking effect, and gait speed as a covariate. This covariate was included based on evidence that gait speed significantly influences gait variability^49,53^. The REML method in JMP Pro Version 13 (SAS Institute Inc., Cary, NC) was used to fit ANOVA models. Prior to analysis, assessments of normality were conducted for each dependent gait measure using quantile plots and goodness-of-fit tests (Shapiro–Wilk). Subsequently, several variables were log-transformed (especially SD and JAV measures) to obtain normally distributed residuals; for ease of interpretation, summary results are presented in the original units. No other substantial deviations from parametric model assumptions were evident. Statistical significance was concluded when *p* < 0.05. Significant interaction effects were examined using simple-effects tests, and *t* tests were used to compare among pairs of conditions. Bonferroni corrections were applied for post hoc paired comparisons among the three load conditions (i.e., $\alpha$ = 0.017). Effect sizes were computed using partial eta squared (${\eta }_{p}^{2}$).

## Results

Outcome measures for both experience groups across all three load conditions are summarized in Tables 2 and 4, and detailed ANCOVA results for the mean gait and GV measures are presented in Tables 3 and 5. Mean stride and step times were significantly longer among the 1st-year vs. the 4th-year groups (Tables 2, 3). Step width differed significantly between load conditions, increasing from the Load_Zero_ and Load_Low_ conditions to the Load_High_ condition.

**Table 2:** Gait measures for each load condition and experience group.

Variable	Load_Zero_		Load_Low_		Load_High_	
	1st	4th	1st	4th	1st	4th
Stride time (s)	1.07 (0.06)	1.04 (0.05)	1.08 (0.07)	1.04 (0.05)	1.09 (0.08)	1.05 (0.05)
Step time (s)	0.54 (0.03)	0.52 (0.03)	0.54 (0.03)	0.52 (0.03)	0.54 (0.04)	0.53 (0.03)
Stride length (m)	1.57 (0.11)	1.56 (0.13)	1.56 (0.12)	1.55 (0.14)	1.53 (0.13)	1.52 (0.14)
Step width (m)	0.10 (0.03)	0.11 (0.03)	0.10 (0.03)	0.11 (0.03)	0.10 (0.03)	0.11 (0.03)

All values reported as means (SDs).

**Table 3:** Analysis of covariance results for mean gait measures.

Variable	Experience (E)			Load (L)			E × L			Gait speed		
	*F*	*p*	${\eta }_{p}^{2}$	*F*	*p*	${\eta }_{p}^{2}$	*F*	*p*	${\eta }_{p}^{2}$	*F*	*p*	${\eta }_{p}^{2}$
Stride time (s)	4.4	**0.040**	0.598	2.7	0.075	0.063	0.1	0.884	0.003	254.6	** < 0.001**	0.693
Step time (s)	5.2	**0.026**	0.566	2.0	0.138	0.053	< 0.1	0.999	< 0.001	215.7	** < 0.001**	0.651
Stride Length (m)	3.7	0.060	0.555	3.0	0.054	0.072	0.2	0.856	0.003	451.9	** < 0.001**	0.761
Step width (m)	2.9	0.096	0.487	4.6	**0.013**	0.066	0.2	0.786	0.006	0.3	0.609	< 0.001

The table presents the *F* statistics, *p-*values, and effect sizes for the main effects of experience level (E: 1st-year vs. 4th-year) and load condition (L: Load_Zero_ vs. Load_Low_ vs. Load_High_), and the interaction effect of experience level and load condition (E × L). The effect of the covariate (gait speed) is also included.

Significant effects are highlighted in bold.

When reporting *p*-values, if the value was smaller than 1 in one thousand, instead of reporting the exact *p*-value up to the first significant decimal figure, we followed standard statistical abbreviation and reported it as *p* < 0.001.

**Table 4:** Gait variability measures for each load condition and experience group.

Variable	Load_Zero_		Load_Low_		Load_High_	
	1st	4th	1st	4th	1st	4th
Stride time SD (s)	21.59 (4.63)	17.83 (3.54)	24.33 (4.46)	19.51 (5.29)	27.94 (10.10)	22.05 (4.97)
Step time SD (s)	12.96 (2.30)	11.20 (2.43)	14.12 (2.22)	11.77 (2.49)	16.48 (4.80)	13.57 (2.50)
Stride length SD (mm)	47.46 (13.65)	42.83 (10.55)	48.17 (12.47)	38.89 (8.05)	52.73 (15.26)	43.20 (9.61)
Step width SD (mm)	33.60 (8.12)	33.66 (6.20)	36.05 (8.00)	36.17 (7.13)	41.99 (9.26)	39.74 (7.21)
Hip JAV (°)	1.26 (0.30)	1.12 (0.15)	1.36 (0.29)	1.22 (0.17)	1.78 (0.50)	1.43 (0.27)
Knee JAV (°)	2.05 (0.53)	1.83 (0.22)	2.28 (0.30)	2.04 (0.29)	2.81 (0.56)	2.42 (0.46)
Ankle JAV (°)	1.50 (0.33)	1.56 (0.36)	1.63 (0.45)	1.68 (0.49)	1.86 (0.44)	1.81 (0.51)
Hip-knee CAV (°)	15.80 (2.54)	14.68 (2.35)	15.93 (2.72)	14.68 (2.19)	18.08 (2.44)	15.70 (2.29)
Knee-ankle CAV (°)	20.72 (2.02)	19.55 (1.31)	20.80 (1.74)	19.99 (1.61)	23.46 (3.45)	21.22 (2.20)
Hip SLE	1.20 (0.12)	1.23 (0.10)	1.26 (0.10)	1.22 (0.10)	1.28 (0.13)	1.25 (0.12)
Knee SLE	1.35 (0.14)	1.32 (0.14)	1.35 (0.13)	1.30 (0.15)	1.29 (0.18)	1.25 (0.18)
Ankle SLE	1.02 (0.14)	0.97 (0.19)	1.08 (0.16)	1.00 (0.20)	1.05 (0.14)	1.03 (0.19)
Hip LLE	0.11 (0.03)	0.12 (0.04)	0.12 (0.03)	0.12 (0.02)	0.11 (0.03)	0.12 (0.04)
Knee LLE	0.11 (0.02)	0.12 (0.03)	0.11 (0.03)	0.11 (0.03)	0.09 (0.03)	0.11 (0.04)
Ankle LLE	0.07 (0.02)	0.07 (0.02)	0.07 (0.02)	0.08 (0.02)	0.05 (0.02)	0.07 (0.02)

All values reported as means (SDs).

**Table 5:** Analysis of covariance results for measures of gait variability (GV).

Variable	Experience (E)			Load (L)			E × L			Gait speed		
	*F*	*p*	${\eta }_{p}^{2}$	*F*	*p*	${\eta }_{p}^{2}$	*F*	*p*	${\eta }_{p}^{2}$	*F*	*p*	${\eta }_{p}^{2}$
**SD**												
Stride time (s)*	13.7	** < 0.001**	0.251	15.7	** < 0.001**	0.187	0.6	0.527	0.015	3.6	0.062	0.032
Step time (s)*	12.2	**0.001**	0.260	22.1	** < 0.001**	0.247	0.8	0.441	0.017	7.0	**0.010**	0.037
Stride length (mm)*	7.7	**0.008**	0.232	4.2	0.018	0.057	1.9	0.150	0.036	0.1	0.785	0.005
Step width (mm)	0.3	0.605	0.007	54.4	** < 0.001**	0.464	1.8	0.176	0.040	5.9	**0.017**	0.001
**JAV**												
Hip (°)*	9.6	**0.003**	0.216	49.1	** < 0.001**	0.445	4.1	**0.019**	0.074	2.1	0.148	0.009
Knee (°)*	8.9	**0.004**	0.224	70.4	** < 0.001**	0.538	1.5	0.233	0.028	2.8	0.100	0.012
Ankle (°)*	< 0.1	0.959	0.001	25.3	** < 0.001**	0.339	0.8	0.467	0.012	0.3	0.571	0.020
**CAV**												
Hip-knee (°)	9.3	**0.004**	0.165	15.1	** < 0.001**	0.160	2.1	0.130	0.045	0.5	0.487	0.016
Knee-ankle (°)*	10.0	**0.003**	0.156	23.8	** < 0.001**	0.274	2.4	0.100	0.043	0.1	0.720	0.002
**SLE**												
Hip	0.4	0.537	0.008	6.1	**0.003**	0.087	3.6	**0.030**	0.066	2.4	0.128	0.002
Knee	< 0.1	0.911	< 0.001	4.5	**0.014**	0.079	0.8	0.448	0.014	0.4	0.520	< 0.001
Ankle	1.0	0.312	0.109	1.4	0.243	0.029	0.3	0.737	0.008	0.1	0.729	0.007
**LLE**												
Hip	0.9	0.337	0.006	0.8	0.473	0.013	1.4	0.242	0.028	10.8	**0.002**	0.025
Knee	3.5	0.068	0.022	3.1	0.048	0.031	1.0	0.372	0.020	4.9	**0.031**	0.032
Ankle	4.5	**0.040**	0.049	2.1	0.132	0.024	1.8	0.168	0.035	6.4	**0.014**	0.024

The table presents the *F* statistics, *p-*values, and effect sizes for the main effects of experience level (E: 1st-year vs. 4th-year) and load condition (L: Load_Zero_ vs. Load_Low_ vs. Load_High_), and the interaction effect of experience level and load condition (E × L). The effect of the covariate (gait speed) is also included.

Significant effects are highlighted in bold.

When reporting *p*-values, if *p*-value was smaller than 1 in one thousand, instead of reporting the exact *p*-value up to the first significant decimal figure, we followed standard statistical abbreviation and reported it as *p* < 0.001.

Both experience level and load condition significantly affected spatiotemporal variability (Table 5). Specifically, the 1st-years had higher variability in stride time, step time, and stride length, versus the 4th-year group. Additionally, variability in stride time, step time, and step width significantly increased with load in both groups (Table 4).

**Figure 2:**
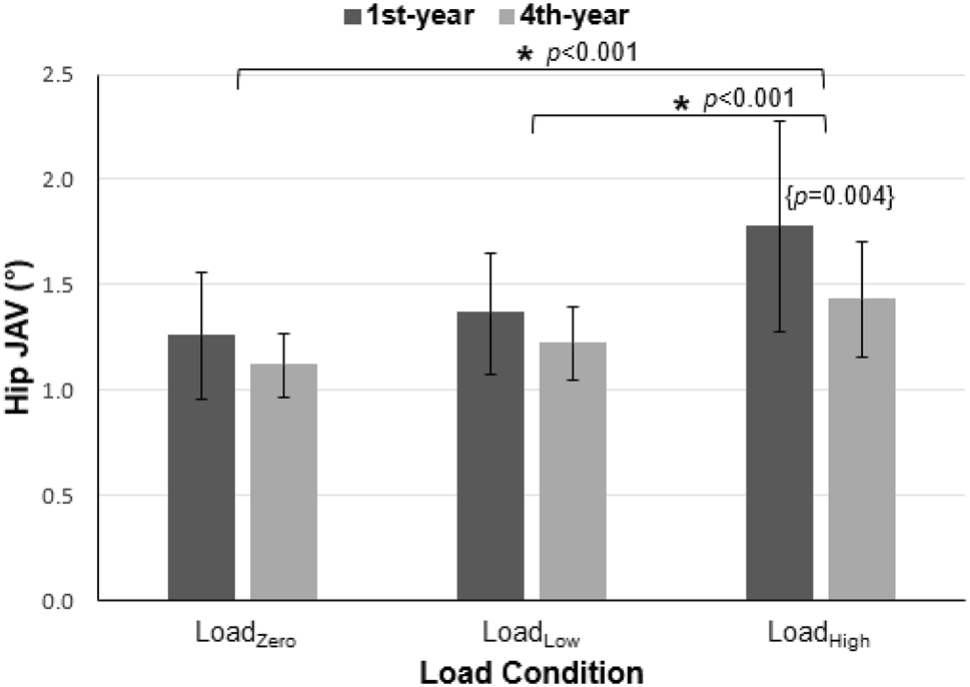
Effects of load and experience level on hip joint angle variability (JAV). Error bars indicate standard deviations. The symbol * indicates a significant paired difference between load conditions, while {} indicates a significant difference between groups.

Hip JAV was significantly affected by the experience × load interaction (Fig. 2). Based on post hoc analyses, an increase with load occurred in both groups, though to a greater extent among the 1st-year cadets. Knee and ankle JAV each increased significantly with load, and knee JAV was significantly higher in the 1st-year vs. the 4th-year groups. Both hip-knee and knee-ankle CAV were significantly higher in the Load_High_ condition, versus the Load_Zero_ and Load_Low_ conditions. The 1st-year group had higher hip-knee CAV and knee-ankle CAV.

Hip SLE significantly increased in the Load_High_ condition compared to the lighter load conditions and was significantly affected by the experience × load interaction (Tables 4, 5). In contrast, knee SLE decreased significantly from the Load_Zero_ condition to the Load_High_ condition. Hip and knee LLE were not significantly affected by either experience level or load condition. There was a significant main effect of experience level on ankle LLE, being higher among the 4th-year vs. the 1st-year groups.

## Discussion

The purpose of this study was to determine the extent to which GV is affected by load carriage and experience, and whether experience-related differences in GV are dependent on load condition. Three categories of GV measures were obtained, which included spatiotemporal variability (SD), joint kinematic variability (JAV and CAV), and Lyapunov exponents (LE). A primary result was that that both experience level and load condition influenced GV measures in all three categories (see Table 5). The less experienced 1st-year group exhibited higher variability in spatiotemporal SDs, JAV, and CAV measures, whereas both groups walked slower and exhibited higher variability with increasing load. The increase in hip JAV with load was greater in the 1st-year group than the 4th-year group. Both groups exhibited enhanced short-term local dynamic stability at the knee joint with increasing load, possibly due to a slower gait speed, while the 4th-year group exhibited less long-term stability at the ankle joint across all load conditions.

The less experienced group had longer stride and step times, and both groups had an increase in step width during the Load_High_ condition. In similar studies involving novices, no changes in mean spatiotemporal measures with different load conditions were found^23,27,54,55^. However, these earlier studies used lighter loads (i.e., 3–20 kg, similar to the current Load_Low_), and differences found here were most substantial during the heavy load condition. In contrast, studies involving more experienced groups and somewhat higher load (i.e., 4–32 kg) have reported conflicting results, potentially due to variations in gait speed between load conditions^7,10^. Participants here were told initially to walk at their typical, preferred pace during load carriage, however they were not given any additional instruction regarding their pace throughout the trials. Gait speed did not change between the Load_Zero_ and Load_Low_ conditions but decreased during the Load_High_ condition in both groups, as expected.

Interestingly, even studies that reported no changes in mean gait measures found significant load effects on measures of GV^23,27,56^. In the current study, step width SD increased with load, but did not differ by level of experience. We also found that stride and step time SD, along with stride length SD, decreased with experience and increased from the Load_Low_ to the Load_High_ conditions. While other studies have investigated the effects of load on the same measures, no significant effects were reported^23,27^. In accordance with findings from studies testing lighter loads^23,27,56^, few significant differences between the Load_Zero_ and Load_Low_ conditions were evident here. Thus, the current results indicate that it is important to consider realistic loads, regularly carried by dismounted soldiers, since changes in spatiotemporal GV may not be linear and may only be exhibited at relatively higher load conditions. Additionally, prior work has shown that increases in spatiotemporal GV (i.e., stride time and length variability) corresponded with an increase in fall risk among older adults^24,25^. However, this result was obtained from a sample different from the highly active, young adults that we tested. Hence, the extent to which such findings of increased GV may transfer to long-term load carriage performance and injury risk are currently unclear.

In terms of joint kinematic variability, JAV at each joint increased with load, and knee JAV was higher in the less experienced group. There was an interaction effect of experience and load on hip JAV, in that hip JAV only increased in the Load_High_ condition and was higher among the less experienced group in this condition. The effect of load carriage on JAV was reported in only one previous study^23^, which found no significant effects with loads of 15% body mass (i.e., ~ 11 kg). Here, CAV for both the hip-knee and knee-ankle joint couples also increased with load and the less experienced group had greater variability. While load carriage research has primarily investigated trunk-pelvis or trunk-thigh coordination, finding a positive relationship between load condition and CAV^22,23^, no other studies to our knowledge have assessed the variability in lower extremity coordination during load carriage. The consistent increase in JAV and CAV we found with increasing load suggests that greater variability reflects a loss of control. Thus, we believe that the less experienced group may have had difficulty in keeping up with the control/performance requirements of the task, as compared to the experienced group, as reflected by increases in JAV and CAV.

LE values, obtained here to quantify local dynamic stability, were consistent with earlier evidence^23,49,50^. Specifically, hip SLE increased with load, though there was a decrease in knee SLE found in the Load_High_ condition among both experience groups. Therefore, short-term local dynamic stability at the hip appeared to be compromised with heavier loads, while stability at the knee improved during the Load_High_ condition even though knee movements were more variable. This positive relationship between short-term local dynamic stability and load implies that the knee joint is primarily responsible for stabilizing the body during load carriage. Additionally, an increase in stability with a heavier load could be a result of participants slowing down to increase control, avoid injury, or minimize fall risk^53^.

LLE was not affected by load condition overall, though the more experienced group exhibited less long-term dynamic stability at the ankle and this difference increased with load. Interestingly, Qiao et al. ^57^ reported a similar finding, that young healthy adults who exhibited higher local dynamic instability were less sensitive to external perturbations while walking, and therefore, may self-regulate their response to balance perturbations. If so, the experienced group here may have purposely allowed for a decreased ankle stability to prepare for perturbations that result from load carriage, since the heaviest load condition requiring the greatest stability was the condition in which long-term ankle stability was decreased. The less experienced group also had decreased ankle stability in the Load_High_ condition, although not to the extent exhibited by the more experienced participants.

The current study was distinct in that previous studies of load carriage have involved treadmill gait, used other methods for constraining gait speed, or tested non-continuous walking^23,57–59^, each of which can reduce the natural gait variability^34,59^. Further, these earlier studies typically captured only 3–5 min of walking, and hence may have derived outcomes that were not representative of prolonged walking (e.g., soldier marches). These studies also may not have had sufficient strides for reliably estimating GV, nor were they conducted using military populations. Nonetheless, our study has some limitations that should be noted. First, while a mean of 92 strides were collected for each subject under all three load conditions, exceeding the recommended number of 50^36^, it remains unknown whether 50 strides is actually sufficient for computing more advanced measures such as the LE^50^. A second limitation is that the loads tested were held constant across participants to be reflective of realistic military conditions, diverging from standardized loads (percent body mass) often reported on in recent literature. This difference in approach may explain discrepancies previously highlighted for some dependent GV measures.

The generalizability of our findings is uncertain: while the 4th-year cadets were relatively more experienced in the load carriage task than those in their first year, dismounted soldiers typically have many years of experience in load carriage, given that they are required to perform this task daily with a variety of loads. Thus, it is unclear to what extent motor skills and strategies continue to improve when cadets transition to becoming soldiers, and how that may affect the relationships studied here. Further, while it is common for soldiers to have recent lower extremity injuries or chronic pain^60^, the current sample only included healthy individuals. In addition to testing a more experienced soldier population, future work should investigate various walking speeds and surfaces since we only evaluated GV at a self-selected pace on a flat surface. Dismounted soldiers must be prepared to perform at a high level under various conditions, which may include time constraints or varying terrains. Thus, additional investigation is needed to confirm the findings presented here are consistent across different extrinsic conditions. Lastly, future studies should investigate the relationship between GV and experience in a variety of tasks, since it is unknown whether the results are also generalizable to more diverse activity types, such as obstacle negotiation.

In summary, we found that measures of gait variability (GV) were more sensitive than more traditional mean measures of gait to differences in experience level and changes in load. The current findings also suggest that experience plays an influential role on the movement strategies used during load carriage gait, implying that experience level should be considered when investigating effective approaches to minimize injury risk and enhance performance. Considering experience level may be especially important for military populations, in which experience varies substantially between new cadets and active-duty soldiers. Finally, the current results emphasize the importance of considering GV measures, as these measures can provide a more in-depth description of an individual’s adaptability and control, highlight alternate movement strategies used in different load conditions, and capture differences related to experience in the gait strategies during load carriage.

## Supplementary Information

Supplementary Information.

## Data Availability

The datasets generated during and/or analyzed during the current study are available from the corresponding author upon reasonable request.
