# Supplementary material for: Task demand and load carriage experience affect gait variability among military cadets
Source: Sci Rep. 2022 Nov 1;12:18347. doi: 10.1038/s41598-022-22881-y (PMC9626617; doi:10.1038/s41598-022-22881-y)
Supplement: Supplementary file 1 — Supplementary Information. [file 41598_2022_22881_MOESM1_ESM.docx]

**Supplementary Methods.** Lyapunov Exponent (LE) Calculations

Prior to calculating LEs (*λ)*, joint angle times series from the hip, knee, and ankle were low-pass ﬁltered using a 2^nd^-order, recursive, Butterworth ﬁlter with a cutoff frequency of 10 Hz (England & Granata, 2007). It was then necessary to reconstruct an appropriate state space, which is any vector space containing a sufficient number of independent coordinates to define the state of the system at any point in time. For this, a single time series from the original data and time-delayed copies can be used (Dingwell, Cusumano, Cavanagh, & Sternad, 2001):

|  | X(𝑡) = [(𝑡), (𝑡 + 𝑇), 𝑥(𝑡 + 2𝑇), …, 𝑥(𝑡 + (𝑛 − 1)𝑇)] | Eq. (S.1) |
| --- | --- | --- |

where *X(t)* is the n-dimensional state vector, *x(t)* are the original data, *T* is the time delay, and *n* is the embedding dimension. Here, *T* was estimated using the first lag when the autocorrelation function reduced to 1 – 1/e of its initial value (Rosenstein, Collins, & De Luca, 1993). The first minimum was chosen, since it provides adjacent delay coordinates with a minimum amount of shared information. Subsequently, the number of state space dimensions, *n*, was chosen based on a Global False-Nearest-Neighbor (GFNN) analysis (Rosenstein, et al., 1993), which compares the distances between neighboring trajectories in the reconstructed state space at successively higher dimensions; this was accomplished by increasing n until the number of false-nearest-neighbors approached zero. False nearest neighbors are defined as sets of points that are very close to each other at dimension n=k but not at n=k+1, which provides a sufficient number of coordinates to define the system state at all points in time (Dingwell, et al., 2001; England & Granata, 2007).

The maximum *λ* (*λ*_1_) was calculated by computing the Euclidean distance between nearest neighbors, *d(t)*, for each data-point in the reconstructed state space *X(t)* across time *t* using (Rosenstein, et al., 1993):

|  | 𝑑(𝑡) = 𝐷𝑒𝜆_1_𝑡 | Eq. (S.2) |
| --- | --- | --- |

where *D* is the initial average displacement between trajectories (*t*=0). However, since experimental time series are finite, a maximum finite-time *λ* must be estimated for each embedded time series by taking the log of both sides:

|  | ln[𝑑_𝑗_(𝑖)] ≈ 𝜆* (𝑖∆𝑡) + ln[𝐷_𝑗_] | Eq. (S.3) |
| --- | --- | --- |

where *d_j_(i)* is the Euclidean distance between the *j*^th^ pair of nearest neighbors after *i* discrete time steps (i.e., *i*Δ*t*). Euclidean distances between neighboring trajectories in state space were then calculated as a function of time and averaged over all original pairs of nearest neighbors, and the maximum finite-time LE, *λ**, was estimated from the slopes of linear fits to curves:

|  | 𝑦(𝑖) = (1/∆𝑡) 〈 ln [𝑑𝑗(𝑖)] 〉 | Eq. (S.4) |
| --- | --- | --- |

where 〈–〉 indicates the average over all value of *j* (Rosenstein, et al., 1993). Estimates of *𝜆** were calculated over two different time scales. Short-term exponents (SLE) were computed between 0 and 1 stride, and long-term exponents (LLE) were computed between 4 and 7 strides (Dingwell, et al., 2001; England & Granata, 2007; Rosenstein, et al., 1993).
